# The Ethiopian National Immunization Technical Advisory Group (E-NITAG): Establishment, Achievements and the Future

**DOI:** 10.4314/ejhs.v34i1.12

**Published:** 2024-01

**Authors:** Yemane Berhane, Telahun Teka, Meseret Zelalem, Yohannes Lakew, Amha Mekasha, Bogale Worku, Genet G/Medhin, Liya Wassie, Liya Wondwossen, Mulat Nigus, Samuel Teshome, Shiferaw Mitiku, Solomon Tessema Memirie, Teferi Fenta, Workeabeba Abebe, Yirgu Gebrehiwot

**Affiliations:** 1 Addis Continental Institute of Public Health, Addis Ababa, Ethiopia; 2 Department of Pediatrics and Child Health, School of Medicine, College of Health Sciences, Addis Ababa University, Ethiopia; 3 Maternal, Child and Adolescent Health Lead Executive Office. Ministry of Health, Ethiopia; 4 Department of Obstetrics & Gynecology, Saint Paul's Hospital Millennium Medical College, Addis Ababa, Ethiopia; 5 Armauer Hansen Research Institute, Addis Ababa, Ethiopia; 6 International Vaccine Institute, Addis Ababa University, Ethiopia; 7 College of Business and Economics, School of Commerce, Department of Logistics and Supply Chain Management, Addis Ababa University, Ethiopia; 8 Addis Center for Ethics and Priority Setting, Addis Ababa University, Ethiopia; 9 School of Pharmacy, College of Health Sciences, Addis Ababa University; 10 Department of Obstetrics and Gynecology, College of Health Sciences, Addis Ababa University, Ethiopia

**Keywords:** Immunization, Vaccines, NITAG, Ethiopia

## Abstract

The National Immunization Program (NIP) was introduced in Ethiopia in 1980. The NIP has expanded the number of vaccines from six to more than 14 in 2023. However, decisions on new vaccine introduction and other vaccine-related matters were not systematically deliberated nationally. Thus, the need to establish a national body to deliberate on vaccine and vaccination matters, in addition to the global immunization advisory groups, has been emphasized in the last decade. This article presents the establishment and achievements of the Ethiopian NITAG. The E-NITAG was established in 2016 and maintained its active role in providing recommendations for new vaccine introduction and improving the delivery of routine vaccines. The external assessment indicated the E-NITAG was highly functional and played a critical role in enhancing the vaccination practice in Ethiopia, especially during the COVID-19 pandemic. The absence of a dedicated secretariat staff was the major bottleneck to expanding the role of the E-NITAG beyond responding to MOH requests. The E-NITAG must be strengthened by establishing a secretariat that can eventually grow as an independent institution to address complex vaccine-related issues the NIP needs to address.

## Introduction

Ethiopia initiated the National Immunization Program (NIP) in 1980. For the first two and half decades, the NIP focused on six childhood immunizations, including BCG, Polio, DPT, and Measles (1). In the last few decades, the NIP has introduced many additional vaccines that targeted priority diseases in children, such as vaccines against Hemophilus influenza type b (Hib), hepatitis B (Hep B), Pneumococcal vaccine (PCV), rotavirus, and more recently, in adolescents and adults, such as vaccines against human papillomavirus (HPV) in adolescent girls and several COVID-19 vaccines that includes adults. Several new vaccines are in the pipeline for introduction into the NIPs of low-income countries. Despite promising efforts to introduce new vaccines, increasing routine immunization coverage remains atop priority (2–4).

Introducing new vaccines into the NIPs is a necessary but complicated process. Decisions require multi-stakeholder engagement at various levels. Some issues are political, economic, and logistical. The Inter-agency Coordinating Committee (ICC) handles these issues, and the final decision falls to the Ministries of Health and Finance. Ensuring vaccine safety nationally requires regulating good manufacturing practices regulated by the Ethiopian Food and Drug Administration (EFDA).

The critical aspect of vaccine introduction that involves examining the scientific evidence of effectiveness, timing, and prioritizing has not been optimally organized nationally before the establishment of the E-NITAG (5). Establishing institutionalized and systematic procedures to justify the introduction of new vaccines and adjust existing vaccines and vaccination schedules and policies has become a priority for many low-income countries, including Ethiopia, leading to the establishment of National Immunization Technical Advisory Groups (NITAGs). The NITAG is a multidisciplinary body of national experts providing evidence-based recommendations on immunization to policy-makers and program managers (6).

## Establishment and Rationale

The Ethiopian NITAG (E-NITAG) was established by the Ministry of Health (MoH), and members with diverse expertise (including public health, child and maternal health, and biomedical sciences) were officially appointed in May 2016. Following the appointment of members by the MoH, the E-NITAG reviewed and enriched its terms of reference (ToR). In addition, all E-NITAG members received extensive orientation on their roles and the procedures they must adopt. The service of the core members is limited to two terms, each term being three years. The World Health Organization (WHO) supported the establishment of the E-NITAG through well-designed procedures (7).

Membership in the Ethiopian NITAG is divided into core and non-core. The MOH appoints core members based on their merit. The selected core members do not represent any organization in their capacity as an E-NITAG member. The non-core members are ex-officio members of the NIP and experts from organizations working on immunization and related stakeholders. The NITAG also invites additional experts on an *ad-hoc* basis for a specific agenda or meeting to enrich deliberations and hear expert opinions. The E-NITAG has elected a chairperson and vice-chairperson. The chairperson ensures that quorum is achieved and conflict of interest and confidentiality agreements are declared and signed by E-NITAG members at every meeting. The NIP serves as the secretariat of the E-NITAG. The expertise in the E-NITAG includes epidemiology, pediatrics, infectious diseases, public health, health economics, immunology, neurology, microbiology, logistics, pharmacology, and vaccinology. E-NITAG members received relevant training on various occasions before or after joining the E-NITAG. The membership criteria and the procedures follow global standards for NITAGs set by the WHO (8).

The Ethiopian NITAG was established to provide unbiased and evidence-based recommendations to the Ministry of Health on using available and newly introduced vaccines. In its review, the E-NITAG often considers vaccine characteristics such as safety and efficacy profiles, disease portfolios, and programmatic capacity to offer recommendations for optimal implementation of national policies on vaccines and immunization program. The E-NITAG recommendations are based on scientific evidence, the local epidemiology of vaccine-preventable diseases, and the implementation capacity of the national immunization program. The invaluable role of NITAGs in strengthening the evidence base in immunization-related decisions was observed in other African countries (9,10).

## The E-NITAG Procedures

The E-NITAG, like many other NITAGs, follows a review and deliberation procedure that considers the rigor of the evidence and the timely delivery of recommendations (11). The timeliness of NITAG recommendations is critical, especially during emergencies such as the COVID-19 pandemic, as vaccines are often in short supply and the urgency to protect the key population in the country is high (12–14).

**Figure 1:**
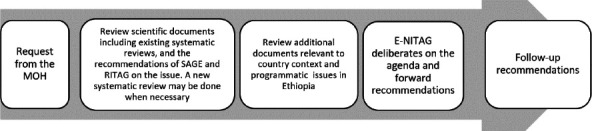
The E-NITAG procedure for making policy recommendations

The E-NITAG is well connected to the Strategic Advisory Group of Experts on Immunization (SAGE) at WHO, the Regional Immunization Technical Advisory Group (RITAG) at WHO-AFRO, and the Global NITAG Network (GNN). These bodies generate extensive scientific evidence available at the NITAG Resource Center (15). The E-NITAG members have attended technical meetings and webinars to gain access to evidence related to vaccines and immunization programs. As indicated in Figure 1, the E-NITAG has routinely adopted a procedure to address immunization-related issues. The E-NITAG commits to doing a new systematic review only when the evidence generated globally and regionally is insufficient to make context-appropriate recommendations. The E-NITAG invites relevant experts to present evidence and explanations as necessary without restriction during its deliberation (11).

## The E-NITAG Achievements

The E-NITAG has maintained a fully functionality status at all times since its establishment; the functionality indicators, according to the WHO, include the existence of a legislative basis, presence of a Terms of Reference, composition of core members in at least five areas, meeting at least two times a year, declaration of conflict of interest, and sharing agenda ahead of meeting with background documents (16,17). The E-NITAG met at least twice per year, more than the WHO NITAG functionality indicators required. The E-NITAG became a member of the GNN in 2017 (18). The E-NTIAG participated in several SAGE and RITAG meetings in person and virtually.

The E-NITAG has successfully and timely provided scientific and practical recommendations on many critical immunization issues since its establishment, including introducing new vaccines into the routine vaccination program and addressing emerging issues such as introducing several COVID-19 vaccines. The coordination and collaboration among various stakeholders during the COVID-19 pandemic were critical in ensuring the introduction of COVID-19 vaccines with unprecedented efficiency. E-NITAG reviewed scientific literature and evidence from countries with stringent regulatory procedures to promptly adopt appropriate COVID-19 vaccines for the country's context (11).

The E-NITAG recommended the introduction of new vaccines or schedules, including HPV, a second dose of measles-containing vaccine (MCV2), HepB-Birth dose, a switch from PCV10 two doses vial to PCV 13 and four doses vial, COVID-19 vaccines, a second dose of inactivated polio vaccine (IPV2), and a switch from TT to Td. In addition, the E-NITAG provided recommendations on implementing measles SIAs during the COVID-19 pandemic, nOPV2 use for cVDPV2 outbreak response, and strengthening the operational conditions of the routine immunization program. The E-NITAG also recommended strengthening surveillance and research relevant to improve evidence-based decisions in the country.

Overall, the E-NITAG played the expected roles in the first two terms. In the second term, membership expanded to include additional relevant expertise. As a result, core membership grew from seven in the first term to thirteen in the second term. The external evaluation by the WHO team indicated that the E-NITAG has maintained a high level of functionality. The commitment of the members to ensure a high level of attendance without any incentive was highly appreciated by the external assessors; the E-NITAG met promptly, and the majority of members attended meetings regularly.

## Challenges

Despite the successful establishment and functionality, the E-NITAG had some constraints. The lack of a fully dedicated secretariat hindered the expansion of its scope. The NIP serving as a secretariat to the E-NITAG had many competing priorities and severe limitations in human resources itself. The country's research on vaccines and immunization is very limited; thus, the E-NITAG had to adopt evidence from the region and other relevant contexts to fulfill its advisory role. Access to surveillance and regulatory data was limited due to a lack of a well-organized secretariat; in addition, the responsible bodies lack mechanisms to share relevant data with stakeholders, including the E-NITAG. The COVID-19 pandemic also created constraints in terms of workload, as the pandemic was evolving rapidly and generating a large volume of information/disinformation. Due to the limitations mentioned above, although the E-NITAG was expected to contribute to improving vaccination practices in general, its role has been limited to responding to requests for technical input from the MOH so far. These constraints were similarly observed in other low-income countries (19).

## The Way Forward

In the future, establishing a well-organized and resourced secretariat must be prioritized. The E-NITAG will likely be needed to address growing opportunities for new vaccines, which accompany technological advances, emerging pathogens, and pandemic threats. The immunization program will likely be challenged more by misinformation, disinformation, and vaccine hesitancy, which a multidisciplinary technical advisory group must address. Strengthening surveillance and research in collaboration with national stakeholders, including universities and research institutes, is also essential. Promoting data sharing and establishing mechanisms for responsible use of available data/evidence must be prioritized. The E-NITAG could eventually grow as an independent institution to address complex immunization-related issues effectively.
